# Contribution of Hepatitis B Virus Infection to the Aggressiveness of Primary Liver Cancer: A Clinical Epidemiological Study in Eastern China

**DOI:** 10.3389/fonc.2019.00370

**Published:** 2019-05-21

**Authors:** Fan Yang, Longteng Ma, Yuan Yang, Wenbin Liu, Jun Zhao, Xi Chen, Mengchao Wang, Hongwei Zhang, Shuqun Cheng, Feng Shen, Hongyang Wang, Weiping Zhou, Guangwen Cao

**Affiliations:** ^1^Department of Epidemiology, Second Military Medical University, Shanghai, China; ^2^Eastern Hepatobiliary Surgery Hospital, Second Military Medical University, Shanghai, China

**Keywords:** primary liver cancer, hepatitis virus, radical resection, prognosis, aggressiveness

## Abstract

**Background and aims:** The contribution of hepatitis B virus (HBV) infection to the aggressiveness of primary liver cancer (PLC) remains controversial. We aimed to characterize this in eastern China.

**Methods:** We enrolled 8,515 PLC patients whose specimens were reserved at the BioBank of the hepatobiliary hospital (Shanghai, China) during 2007–2016. Of those, 3,124 who received primary radical resection were involved in survival analysis. A nomogram was constructed to predict the survivals using preoperative parameters.

**Results:** Hepatocellular carcinoma (HCC), intrahepatic cholangiocarcinoma (ICC), and combined hepatocellular cholangiocarcinoma (CHC) accounted for 94.6, 3.7, and 1.7%, respectively. The rates of HBV infection were 87.5, 49.2, and 80.6%, respectively. HBV infection was significantly associated with 10-year earlier onset, more cirrhosis, higher α-fetoprotein, higher carbohydrate antigen 19-9 (CA19-9), more microvascular invasion (MVI), lower neutrophil-to-lymphocyte ratio (NLR), and lower platelet-to-lymphocyte ratio (PLR) in HCC. HBV infection was also associated with 7-year earlier onset, more cirrhosis, higher α-fetoprotein, more MVI, and lower PLR in ICC. In the multivariate Cox analysis, high circulating HBV DNA, α-fetoprotein, CA19-9, NLR, tumor size, number, encapsulation, Barcelona Clinic Liver Cancer (BCLC) stage, and MVI predicted an unfavorable prognosis in HCC; only CA19-9 and BCLC stage, rather than HBV-related parameters, had prognostic values in ICC. A nomogram constructed with preoperative HBV-related parameters including HBV load, ultrasonic cirrhosis, and α-fetoprotein perform better than the current staging systems in predicting postoperative survival in HCC.

**Conclusion:** HBV promotes the aggressiveness of HCC in Chinese population. The contributions of HBV to ICC and other etiological factors to HCC might be indirect *via* arousing non-resolving inflammation.

## Introduction

Primary liver cancer (PLC), comprising hepatocellular carcinoma (HCC, 70–90%), intrahepatic cholangiocarcinoma (ICC, 10–20%), and rare histotypes including combined hepatocellular cholangiocarcinoma (CHC), is the second leading cause of cancer death in men and the sixth leading cause of cancer death in women worldwide (1, 2). The incidence rates of PLC remain highest in Asia. China alone accounts for half of global PLC (1). Over decades, the mortalities increased in Europe and America and decreased in East Asia (1).

Of global PLC, 56% were attributable to hepatitis B virus (HBV) and 20% to hepatitis C virus (HCV) (3). Chronic HBV infection is the major cause of HCC in Asian and African countries (4). Although HCV infection is the leading cause of HCC in most European and American countries, the contribution of HBV is increasing possibly because of immigration. Aflatoxin B1 exposure, alcohol consumption, sugar consumption, and diabetes also contribute to the development of HCC (1–5). Aflatoxin B1 exposure or smoking increases the occurrence of HCC caused by other factors (6, 7). Infection with *Opisthorchis viverrini* and *Clonorchis sinensis*, hepatolithiasis, and primary sclerosing cholangitis are associated with cholangiocarcinoma (8). Chronic infection with HBV or HCV also increase the risk of ICC (9, 10). However, it remains to be identified whether the risk factors promote the development of HCC or ICC directly or indirectly *via* inducing inflammation.

The association of etiological factors and PLC prognosis remains controversial. Data from Australia and the United States indicate that HBV-related HCC has a better prognosis than HCV-related HCC, which is hardly repeated in other populations (11–15). Thus, we performed this large epidemiological study to clarify the contribution of HBV infection to the aggressiveness of major PLC histotypes.

## Materials and Methods

### Patient Enrollment

From January 1st 2007 to March 31st 2016, 8,515 consecutive PLC patients who received hepatectomy at the Eastern Hepatobiliary Surgery Hospital (Shanghai, China) and had their removed tissues reserved in the BioBank were enrolled in this study. Their diagnoses were pathologically confirmed. Radical resection was defined as follows: (i) complete resection of all tumor nodules, with the cut surface free of cancer cells by pathologic examination; (ii) no macroscopic tumor thrombosis in the portal vein (main trunk or two major branches), hepatic veins, or bile duct; (iii) number of tumor nodules not exceeding three; (iv) serum α-fetoprotein (AFP), if positive, declined to undetectable level 2 months after surgery; and (v) no extrahepatic metastasis. Six months after surgical treatment, patients were regularly followed up through telephone by the same team of professional staff, followed by another five sequential follow-ups at the time point of 1, 2, 3, 4, and 5 years after surgery. In the telephone interview, we collected data including survival situation, treatment(s) received after surgery, as well as the exact date of death in case of death. The final date of follow-up was August 31st, 2017. Patients who survived were censored at their last follow-up. Patients with microvascular invasion (MVI) were recommended to receive postoperative transcatheter arterial chemoembolization (TACE) as previously described (16). Patients with imaging evidence of tumor recurrence were recommended to receive second resection or radiofrequency ablation (RFA) (17).

### Data Collection

Demographical information, pathological examinations (including nodule number, capsule integrity of tumor, and MVI), and results of the latest pre-operative laboratory examinations [including serum AFP, carbohydrate antigen 19-9 (CA19-9), HBV parameters, routine blood test, and liver function tests] were extracted from electronic medical records. Child–Pugh score and Barcelona Clinic Liver Cancer (BCLC) stage were determined as previously described (18, 19).

### Statistical Analysis

HBV infection was defined if a patient was seropositive for HBsAg and/or HBV DNA (20). Occult HBV infection (OBI) was defined as the presence of HBV DNA in a patient seronegative for HBsAg (21). Neutrophil–lymphocyte ratio (NLR) and platelet–lymphocyte ratio (PLR) were calculated as neutrophil count and platelet count divided by lymphocyte count, respectively. Their cutoff values were determined using X-tile software (http://www.tissuearray.org/rimmlab/xtile.html, RRID: SCR_005602). Cutoff values of AFP, CA19-9, total bilirubin, direct bilirubin, and albumin were the same as those in previous studies (22, 23). Categorical variables including the positive rates of hepatitis B surface antigen (HBsAg) were compared by χ^2^ test or Fisher's exact test when appropriate. Continuous variables with skewed distribution were compared by Kruskal–Wallis ANOVA for multiple group comparison and Mann–Whitney *U*-test for double group comparison. The Bonferroni correction was applied for multiple comparisons. The Kaplan–Meier method was applied to estimate overall survival (OS), and the log-rank test was performed to compare the difference between survival curves. A Cox proportional hazard model was applied to calculate the hazard ratio (HR) and its 95% confidence interval (CI) for each variable. Significant variables in the univariate Cox analysis were introduced into the multivariate Cox model to determine the factors that independently contributed to postoperative survival. Our cohort was randomly dichotomized into a training cohort and a validation cohort. A Cox model utilizing pre-operatively available variables was fitted in training cohort. A final model was selected by a backward stepwise selection procedure following the Akaike information criterion (24). A nomogram was formulated by applying the *rms* package in R (25). The performance of nomogram was measured by the concordance index (C-index) and calibration plots with 1,000 bootstraps. Comparisons of the prediction power between the nomogram and independent prognostic factors or clinical staging systems were performed using the rcorrp.cens package in Hmisc in R and were evaluated by the C-index (26). The accuracy of the nomogram was validated in the validation cohort. All statistical analyses were two-sided and performed using SPSS V21.0 for Windows (http://www-01.ibm.com/software/uk/analytics/spss/, RRID: SCR_002865) and RStudio V3.9.2 (http://www.rstudio.com/RRID:SCR_000432). *P* < 0.05 was considered as statistically significant.

## Results

Patients were from almost all provinces of mainland China except Tibet. Patients from eastern China accounted for 93.9% (Supplementary Figure 1). They are self-reported Chinese with a median age of 53 years [interquartile range (IQR), 46–61 years]. Patients were predominantly male, with a male-to-female ratio of 6.15. Of the 8,515 patients, 8,056 (94.6%) had HCC, 314 (3.7%) had ICC, and 145 (1.7%) had CHC. The proportions of patients seropositive for HBsAg, HBV DNA, and anti-HCV antibody were 87.3, 51.7and 1.7% in HCC, 49.2, 33.8, and 1.8% in ICC, and 80.6, 41.5, and 1.4% in CHC, respectively. OBI accounted for 0.2% in HCC. Compared with ICC patients, HCC patients had a higher male-to-female ratio, higher proportions of AFP positivity, HBsAg positivity, and HBV DNA positivity, and a lower proportion of CA19-9 positivity. Compared with HCC patients, CHC patients had higher proportions of CA19-9 seropositivity, NLR (>3.3), and PLR (>117). These data are shown in Supplementary Table 1.

### Demographical and Clinical Characteristics Between Primary Liver Cancer Patients With Hepatitis B Virus Infection and Those Without Hepatitis B Virus Infection

Compared with HCC patients without HBV infection, HCC patients with HBV infection were 10 years younger and had higher proportions of positive AFP (≥20 ng/ml), positive CA19-9 (≥37 U/ml), the presence of liver cirrhosis, high direct bilirubin (>7 μmol/L), advanced BCLC stage, and the presence of MVI and lower proportions of NLR (>3.3) and PLR (>117; Table 1). Compared with ICC patients without HBV infection, those with HBV infection were 7 years younger and had a higher male-to-female ratio, higher proportions of positive AFP, cirrhosis, and MVI and lower proportions of PLR (>117) and advanced Child–Pugh score (B vs. A; Table 2). Similarly, CHC patients with HBV infection were 9 years younger and had higher proportions of AFP positivity and cirrhosis, and lower proportions of NLR (>3.3) and PLR (>117) than those without HBV infection (Supplementary Table 2).

**Table 1:** Comparison of demographical and clinical characteristics between HCC patients with HBV infection and those without HBV infection.

**Variable**	**Patients without HBV infection (*N* = 1,010)^a^**	**Patients with HBV infection (*N* = 6,976)^a^**	***P*^b^**
**Gender**			
Female	139 (13.8)	916 (13.1)	0.580
Male	871 (86.2)	6,060 (86.9)	
**Age**			
Medium (IQR)	62 (54–68)	52 (45–60)	<0.001
≤40	50 (5.0)	825 (11.9)	
40–60	404 (40.0)	4,526 (65.2)	
>60	556 (55.0)	1,586 (22.9)	
**Cirrhosis (ultrasound)**			
No	788 (82.9)	3,518 (53.4)	<0.001
Yes	163 (17.1)	3,071 (46.6)	
**Cirrhosis (pathology)**			
No	767 (76.2)	3,559 (51.2)	<0.001
Yes	239 (23.8)	3,393 (48.8)	
**AFP (ng/mL)**			
Negative (<20)	477 (47.9)	2,389 (34.7)	<0.001
Positive (≥20)	519 (52.1)	4,495 (65.3)	
**CA19-9 (U/mL)**			
Negative (<37)	873 (89.6)	5,251 (79.3)	<0.001
Positive (≥37)	101 (10.4)	1,373 (20.7)	
**HBeAg**			
Negative	1,007 (99.7)	4,880 (70.3)	<0.001
Positive	3 (0.3)	2,059 (29.7)	
**HBcAb**			
Negative	144 (14.3)	1 (0.0)	<0.001
Positive	866 (85.7)	6,936 (100.0)	
**HBV DNA (copies/mL)**			
Undetectable (<500)	849 (100.0)	2,812 (41.5)	<0.001
Detectable (≥500)	0 (0)	3,957 (58.5)	
**Total bilirubin (μmol/L)**			
≤20	846 (85.4)	5,700 (83.6)	0.150
>20	145 (14.6)	1,121 (16.4)	
**Direct bilirubin (μmol/L)**			
≤7	786 (79.3)	5,183 (76.0)	0.021
>7	205 (20.7)	1,638 (24.0)	
**Albumin (g/L)**			
>35	951 (96.9)	6,362 (95.1)	0.009
≤35	30 (3.1)	330 (4.9)	
**NLR**			
≤3.3	813 (80.4)	5,742 (83.0)	0.042
>3.3	198 (19.6)	1,176 (17.0)	
**PLR**			
≤117	541 (53.6)	4,540 (65.3)	<0.001
>117	468 (46.4)	2,417 (34.7)	
**Child–pugh score**			
A	920 (98.9)	6,333 (98.9)	0.991
B	11 (1.1)	72 (1.1)	
**BCLC stage**			
0	29 (2.9)	334 (4.8)	<0.001
A	326 (32.6)	2,575 (37.2)	
B	526 (52.5)	2,920 (42.2)	
C	120 (12.0)	1,093 (15.8)	
**Tumor diameter (cm)**			
<3	149 (14.9)	1,518 (22.0)	<0.001
≥3	848 (85.1)	5,393 (78.0)	
**Tumor number**			
Single	858 (86.1)	5,560 (80.5)	<0.001
Multiple	138 (13.9)	1,345 (19.5)	
**Tumor encapsulation**			
No	232 (23.1)	1,689 (24.4)	0.364
Yes	771 (76.9)	5,220 (75.6)	
**MVI**			
No	670 (66.9)	4,297 (62.0)	0.003
Yes	332 (33.1)	2,632 (38.0)	

a *Data are presented as number (%), unless otherwise indicated. Some data do not sum up to the total number for the existence of missing data*.

b *For age (continuous variable) and BCLC stage (rank variable), Mann–Whitney U test was conducted. For other variables (categorical variables), chi-square test was conducted*.

*HCC, Hepatocellular carcinoma; HBV, hepatitis B virus; IQR, interquartile range; AFP, α-fetoprotein; CA19-9, carbohydrate antigen 19-9; HBeAg, hepatitis B e antigen; HBcAb, hepatitis B core antibody; NLR, neutrophil-lymphocyte ratio; PLR, platelet-lymphocyte ratio; BCLC, Barcelona Clinic Liver Cancer; MVI, microvascular invasion*.

**Table 2:** Comparison of demographical and clinical characteristics between ICC patients with HBV infection and those without HBV infection.

**Variable**	**Patients without HBV infection (*N* = 157)^a^**	**Patients with HBV infection (*N* = 152)^a^**	***P*^b^**
**Gender**			
Female	72 (45.9)	27 (17.8)	< 0.001
Male	85 (54.1)	125 (82.2)	
**Age**			
Medium (IQR)	61 (56–68)	54 (47–61)	< 0.001
≤40	5 (3.2)	8 (5.3)	
40–60	65 (41.4)	105 (69.1)	
>60	87 (55.4)	39 (25.7)	
**Cirrhosis (ultrasound)**			
No	135 (91.2)	78 (54.5)	< 0.001
Yes	13 (8.8)	65 (45.5)	
**Cirrhosis (pathology)**			
No	143 (92.9)	83 (54.6)	< 0.001
Yes	11 (7.1)	69 (45.4)	
**AFP (ng/mL)**			
Negative (<20)	138 (89.6)	98 (65.3)	< 0.001
Positive (≥20)	16 (10.4)	52 (34.7)	
**CA19-9 (U/mL)**			
Negative (<37)	71 (46.4)	77 (54.2)	0.180
Positive (≥37)	82 (53.6)	65 (45.8)	
**HBeAg**			
Negative	157 (100.0)	107 (70.4)	< 0.001
Positive	0 (0.0)	45 (29.6)	
**HBcAb**			
Negative	41 (26.1)	0 (0.0)	< 0.001
Positive	116 (73.9)	162 (100.0)	
**Total bilirubin (μmol/L)**			
≤20	140 (90.3)	126 (84.6)	0.129
>20	15 (9.7)	23 (15.4)	
**Direct bilirubin (μmol/L)**			
≤7	130 (83.9)	118 (79.2)	0.293
>7	25 (16.1)	31 (20.8)	
**Albumin (g/L)**			
>35	148 (96.7)	137 (97.2)	1.000
≤35	5 (3.3)	4 (2.8)	
**NLR**			
≤3.3	101 (64.3)	100 (65.8)	0.788
>3.3	56 (35.7)	52 (34.2)	
**PLR**			
≤117	65 (41.4)	91 (59.9)	0.001
>117	92 (58.6)	61 (40.1)	
**Child–pugh score**			
A	146 (95.4)	141 (100.0)	0.029
B	7 (4.6)	0	
**BCLC stage**			
0	1 (0.7)	2 (1.3)	0.573
A	35 (23.0)	28 (18.8)	
B	80 (52.6)	82 (55.0)	
C	36 (23.7)	37 (24.8)	
**Tumor diameter (cm)**			
<3	8 (5.3)	16 (10.9)	0.079
≥3	142 (94.7)	131 (89.1)	
**Tumor number**			
Single	128 (85.3)	119 (81.0)	0.313
Multiple	22 (14.7)	28 (19.0)	
**Tumor encapsulation**			
No	135 (90.0)	120 (81.6)	0.039
Yes	15 (10.0)	27 (18.4)	
**MVI**			
No	134 (87.0)	108 (73.0)	0.002
Yes	20 (13.0)	40 (27.0)	

a *Data are presented as number (%), unless otherwise indicated. Some data do not sum up to the total number for the existence of missing data. Some percentages do not sum up to 100 because of rounding*.

b *For age (continuous variable) and BCLC stage (rank variable), Mann–Whitney U test was conducted. For other variables (categorical variables), chi-square test was conducted*.

### Postoperative Survival

Patients who received first radical resection at the study hospital (*n* = 5,602) were invited to join in the survival analysis. Of those, 2,478 (1,932 refused to be followed-up and 546 were lost in the follow-up) were excluded from survival analysis. The remaining 3,124 patients were included in survival analysis (Supplementary Figure 2). The median follow-up time was 1.18 years, with an IQR of 0.78–2.35 years. Supplementary Table 3 shows the baseline characteristics of patients involved in survival analysis and those not involved. Of the 3,124 patients, 1,443 died of this malignancy during follow-up, with the 1-, 3-, and 5-year survival rates of 79.7, 47.5, and 28.6%, respectively. Postoperative 1-, 3-, and 5-year survival rates of patients with each histotype are shown in Supplementary Table 4. Multivariate Cox regression analysis indicated that serum HBV DNA (≥500 copies/ml), AFP (>20 ng/ml), CA19-9 (>37 U/ml), NLR (>3.3), tumor size (≥3 cm in diameter), multiple tumor nodules, incomplete tumor capsule, later more advanced BCLC stage, and MVI independently predicted shorter OS in HCC. Second resection and RFA independently improved OS (Table 3). CA19-9 (>37U/ml), multiple tumor nodules, and BCLC were significantly associated with shorter OS in univariate Cox analysis, while CA19-9 and more advanced BCLC stage were independently associated with OS in ICC (Supplementary Table 5). To further clarify the effect of HBV parameters on the aggressiveness of HCC, multivariate Cox regression analysis was conducted in HBV-positive HCC patients. It was found that HBV DNA (≥500 copies/ml) was significantly associated with shorter OS (Supplementary Table 6), indicating that HCC patients with active HBV replication had shorter OS than those with inactive HBV replication.

**Table 3:** Univariate and multivariate Cox regression analysis of prognostic factors for postoperative survival in HCC patients.

**Variable**	**No. (%) of participants (*n* = 2,963)^a^**	**Univariate analysis HR (95% CI)**	***P***	**Multivariate analysis HR (95% CI)^b^**	***P***
**Gender**					
Female	382 (12.9)	1			
Male	2,581 (87.1)	1.17 (0.98–1.41)	0.080		
**Age**					
<40	324 (10.9)	1			
40–59	1,800 (60.7)	0.85 (0.70–1.03)	0.848		
≥60	839 (28.3)	0.81 (0.66–1.00)	0.814		
**Cirrhosis (ultrasound)**					
No	1,602 (57.6)	1			
Yes	1,181 (42.4)	1.10 (0.97–1.24)	0.136		
**Cirrhosis (pathology)**					
No	1,643 (55.5)	1			
Yes	1,319 (44.5)	1.08 (0.96–1.21)	0.211		
**HBV DNA (copies/mL)**					
<500	1,433 (50.6)	1		1	
≥500	1,397 (49.4)	1.55 (1.38–1.75)	< 0.001	1.35 (1.18–1.55)	< 0.001
**AFP (ng/mL)**					
≤20	1,092 (37.4)	1		1	
>20	1,826 (62.6)	2.03 (1.78–2.32)	< 0.001	1.68 (1.45–1.95)	< 0.001
**CA19-9 (U/mL)**					
≤37	2,284 (81.4)	1		1	
>37	521 (18.6)	1.34 (1.16–1.54)	< 0.001	1.25 (1.07–1.47)	0.005
**HBsAg**					
Negative	382 (13.1)	1			
Positive	2,543 (86.9)	1.34 (1.12–1.61)	0.002		
**HBeAg**					
Negative	2,170 (74.2)	1			
Positive	755 (25.8)	1.21 (1.07–1.38)	0.004		
**Anti-HCV**					
Negative	2,769 (98.1)	1			
Positive	54 (1.9)	0.64 (0.40–1.01)	0.058		
**Total bilirubin (μmol/L)**					
≤20	2,467 (85.5)	1			
>20	417 (14.5)	1.16 (0.98–1.36)	0.078		
**Direct bilirubin (μmol/L)**					
≤7	2,237 (77.6)	1			
>7	647 (22.4)	1.17 (1.02–1.34)	0.027		
**Albumin (g/L)**					
>35	2,707 (94.4)	1			
≤35	161 (5.6)	1.31 (1.04–1.66)	0.023		
**NLR**					
≤3.3	2,477 (83.6)	1		1	
>3.3	485 (16.4)	1.55 (1.34–1.79)	< 0.001	1.42 (1.20–1.68)	< 0.001
**PLR**					
≤117	1,915 (64.7)	1			
>117	1,047 (35.3)	1.41 (1.25–1.59)	< 0.001		
**Tumor diameter (cm)**					
<3	665 (22.6)	1		1	
≥3	2,282 (77.4)	2.20 (1.85–2.62)	< 0.001	1.37 (1.08–1.74)	0.010
**Tumor number**					
Single	2,339 (79.4)	1		1	
Multiple	608 (20.6)	1.69 (1.48–1.93)	< 0.001	1.28 (1.10–1.48)	0.002
**Tumor encapsulation**					
No	523 (17.7)	1		1	
Yes	2,440 (82.3)	0.63 (0.55–0.72)	< 0.001	0.63 (0.54–0.73)	< 0.001
**Child–pugh score**					
A	2,665 (99.0)	1			
B	28 (1.0)	1.98 (1.23–3.20)	0.005		
**BCLC stage**					
0&A	1,355 (46.0)	1		1	
B	1,592 (54.0)	2.15 (1.89–2.43)	< 0.001	1.54 (1.30–1.82)	< 0.001
**MVI**					
No	2,037 (69.1)	1		1	
Yes	911 (30.9)	2.07 (1.83–2.33)	< 0.001	1.66 (1.46–1.90)	< 0.001
**Post-operative tace**					
No	1,359 (45.9)	1			
Yes	1,604 (54.1)	1.20 (1.06–1.35)	0.003		
**Reoperation**					
No	2,764 (93.3)	1		1	
Yes	199 (6.7)	0.49 (0.38–0.64)	< 0.001	0.48 (0.36–0.65)	< 0.001
**Post-operative RFA**					
No	2,716 (91.7)	1		1	
Yes	247 (8.3)	0.67 (0.54–0.82)	< 0.001	0.67 (0.54–0.84)	< 0.001

a *Some data do not sum up to the total number for the existence of missing data. Some percentages do not sum up to 100 because of rounding*.

b *The final model selection was carried out by a backward stepwise selection procedure with the Akaike information criterion. Only significant (P < 0.05) covariates in univariate analysis were included*.

*CI, confidence interval; HR, hazard ratio; TACE, transcatheter arterial chemoembolization; RFA, radiofrequency ablation*.

### Predication for Postoperative Prognosis Using Preoperative Parameters

**Figure 1:**
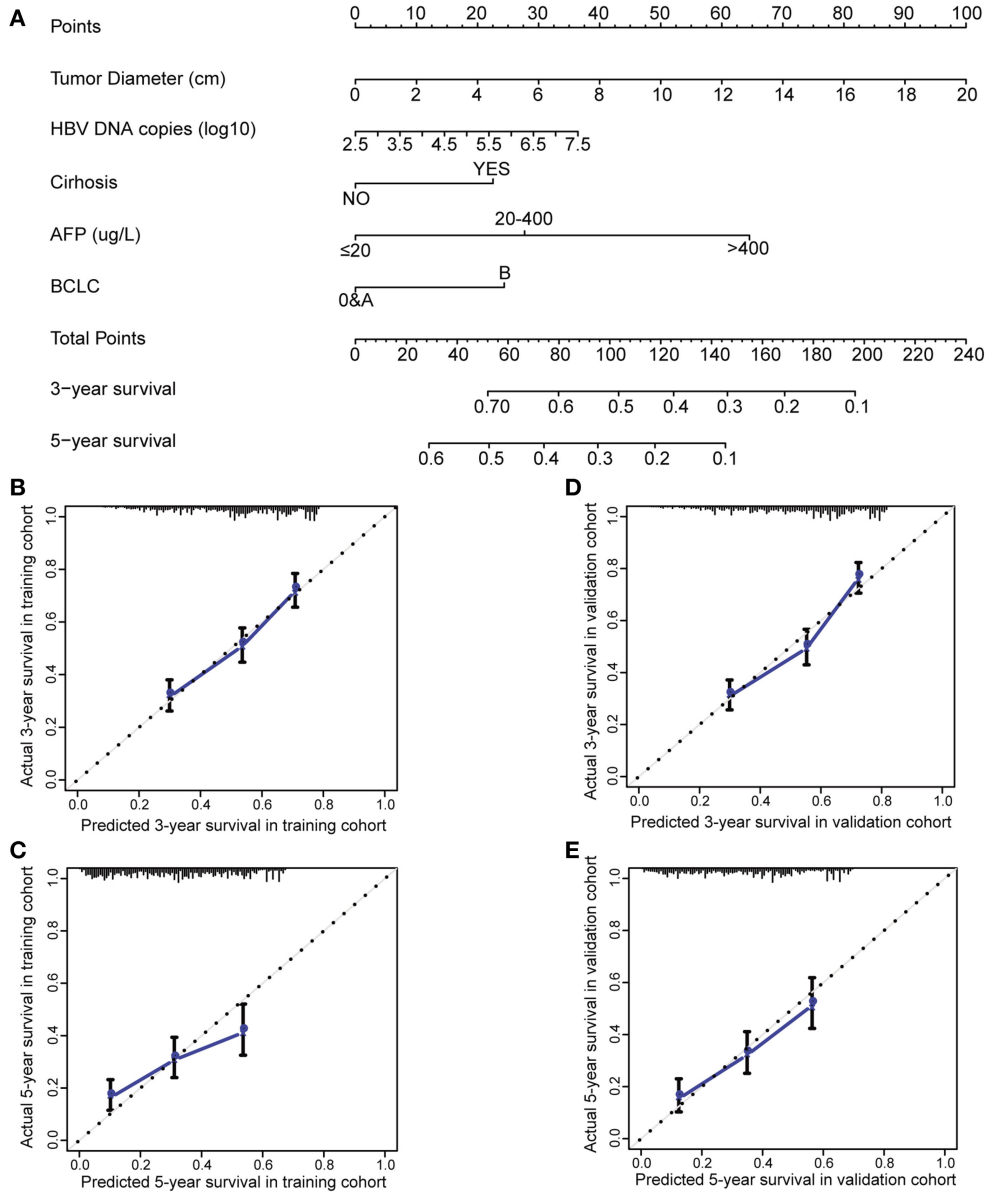
Preoperative nomogram and the calibration curve for predicting postoperative survival of patients with HCC. **(A)** The nomogram. To use this nomogram, a patient's value is located on each variable axis, and a line represents the number of points received for each variable value. The sum of these numbers is located on “Total Points” axis, and a line is drawn downward to the survival axes to determine the likelihood of postoperative 3- or 5-year survival. **(B)** The calibration curve for predicting postoperative 3-year survival in the training cohort. **(C)** The calibration curve for predicting postoperative 5-year survival in the training cohort. **(D)** The calibration curve for predicting postoperative 3-year survival in the validation cohort. **(E)** The calibration curve for predicting postoperative 5-year survival in the validation cohort. AFP, alpha-fetoprotein; BCLC, Barcelona Clinic Liver Cancer; HCC, hepatocellular carcinoma.

To evaluate if HBV-related clinical parameters harvested preoperatively could predict postoperative prognosis, we developed a nomogram using the independent prognostic factors. HCC patients with radical resection (*n* = 2,963) were randomly dichotomized into the training cohort (*n* = 1,482) and validation cohort (*n* = 1,481). All demographical and clinical characteristics were balanced between the training cohort and validation cohort except CA19-9 (Supplementary Table 7). Multivariate Cox analysis in the training cohort showed that preoperative ultrasound cirrhosis, AFP, BCLC stage, HBV DNA, and tumor size were independently associated with OS (Supplementary Table 8). A nomogram that integrated all independent prognostic factors in the training cohort is shown in Figure 1A. The C-index for survival prediction of the nomogram in the training cohort was 0.699 (95% CI, 0.669–0.729). The calibration plot for the probability of postoperative OS showed good agreement between the prediction by nomogram and actual observation (Figures 1B,**C**). The results were faithfully replicated in the validation cohort. The C-index in the validation cohort was 0.700 (95% CI, 0.670–0.730), and a calibration curve showed a good agreement between prediction and actual observation in the probability of 3- and 5-year survivals (Figures 1D,**E**). The C-index was 0.644, 0.638, 0.597, and 0.546 by tumor size, AFP, MVI, and incomplete tumor capsule, respectively, which were significantly lower than that by the nomogram (*P* < 0.001 for each comparison). We then compared the accuracy between the nomogram and each of the clinical staging systems including American Joint Committee on Cancer (AJCC) Staging Manual, 7th ed., Okuda, Chinese University Prognostic Index (CUPI), Groupe d'Etude et de Traitement du Carcinome Hepatocellulaire Prognostic classification (GETCH), and BCLC (16, 27–31). The AJCC 7th, Okuda, CUPI, GETCH, and BCLC systems showed good stratification for the postoperative prognosis of HCC patients in both the training cohort and the validation cohort (Supplementary Figure 3). In the training cohort, the C-index of the nomogram was significantly higher than the AJCC 7th (0.644, *P* < 0.001), Okuda (0.564, *P* < 0.001), CUPI (0.514, *P* < 0.001), GETCH (0.611, *P* < 0.001), and BCLC (0.608, *P* < 0.001). Thus, the nomogram resulted in more accurate prediction for postoperative prognosis of HCC than the prevailing prognostic factors and well-established clinical staging systems.

## Discussion

In this study, we found that HBV infection was associated with 10-year earlier onset and higher proportions of positive AFP, positive CA19-9, the presence of liver cirrhosis, high direct bilirubin, advanced BCLC stage, and the presence of MVI in HCC. AFP, whose expression can be driven by HBV X protein, plays a critical role in promoting the stemness of HCC cells (32). Liver cirrhosis represents anti-inflammatory immune responses to hepatitis B flares or hepatic injuries caused by other chronic inflammation (33). HBV, especially its evolved forms generated during chronic infection and its integrated forms, directly promotes the development of HCC (34–36). This may explain why HBV-related HCC occurs 10 years earlier and is more aggressive than HCC caused by other etiological factors. HBV was inversely associated with NLR and PLR, the well-established inflammatory factors (37, 38), indicating that the non-HBV etiological factors may cause HCC *via* inducing non-resolving inflammation. Interestingly, the HBV-related parameters including HBV DNA, AFP, CA19-9, BCLC stage, and MVI predicted an unfavorable postoperative prognosis independently. Furthermore, the nomogram constructed with HBV-related parameters harvested preoperatively including HBV DNA, liver cirrhosis, AFP, and BCLC stage accurately predicted an unfavorable postoperative prognosis. The prediction power is better than the current clinical staging systems. These evidences indicate that HBV infection promotes the aggressiveness of HCC, at least in the HBV endemic areas. The non-HBV etiological factors promote the development of HCC possibly *via* inducing non-resolving inflammation.

Surprisingly, HBV infection was also associated with 7-year earlier onset, more cirrhosis, higher AFP, more MVI, and lower PLR and Child–Pugh score in ICC. We believe that the changes in these clinical parameters in ICC reflect the role of HBV in generating inflammatory background from which ICC develops, rather than direct etiological role of HBV in ICC. Compared to HCC patients, ICC patients had significantly lower proportions of positive HBsAg, positive HBV DNA, the presence of liver cirrhosis, positive AFP, and the presence of MVI and lower male-to-female ratio, the HBV-related parameters. By contrast, ICC patients had higher proportions of NLR (>3.3) and PLR (>117). These data indicate that HBV-caused inflammation, rather than HBV itself, play a major role in inducing ICC in HBV-infected subjects. CA19-9 and BCLC stage were not associated with HBV infection in ICC, but they predicted an unfavorable prognosis in ICC independently. These data indicate that HBV infection is not related to the aggressiveness of ICC. HBV promotes the development of ICC indirectly *via* inducing non-resolving inflammation. Antiviral treatment reduces the risk of ICC (9, 10), possibly *via* reducing the non-resolving inflammation caused by active HBV infection.

HBV infection accounted for 87.5% of HCC, while HCV infection only accounted for 1.7%. This difference might be fundamentally related to the genetic predispositions. The genotypes and/or allele of *HLA-DQ, HLA-DP*, and *NFKBIA* single-nucleotide polymorphisms (SNPs) that significantly increased the risk of chronic HBV infection are more frequent in Chinese population than in European population (http://www.hapmap.org/) (39–42). These genetic predispositions in Chinese population facilitate chronic transformation of HBV infection, possibly *via* weakening the corresponding antiviral immune function. HBV that evolved in chronic inflammatory microenvironment promotes the occurrence and aggressiveness of HCC. Instead, the C/C genotype of a SNP (rs12979860) of the *IL28B* gene, which is strongly associated with spontaneous clearance of HCV, is more frequent in Chinese population than in African or European population (http://www.hapmap.org/) (43). Thus, HCV might be more apt to cause HCC-inducing non-resolving inflammation in Western populations than in Chinese population. Non-resolving inflammation caused by chronic HCV infection might promote the aggressiveness of HCC, especially in Western populations.

Our study has several limitations. First, selection bias cannot be avoided in a single center. Second, severity of cirrhosis, family history, exposure to aflatoxin, metabolic syndrome, dietary changes, alcohol consumption, and cigarette smoking were not included because these data were not intact in their medical records. Third, compared with patients lost to follow-up, patients who were successfully followed-up had higher AFP level, higher Child–Pugh score, higher BCLC stage, larger tumor diameter, lower albumin level, larger tumor diameter, higher proportion of multiple tumor nodules, tumor encapsulation, and MVI, most of which were associated with shorter OS in HCC, so the survival rates of HCC should be underestimated. Fourth, the effects of postoperative radiotherapy, chemotherapy, stereotactic radiation, percutaneous ethanol injection, antiviral treatment, and targeted therapy as well as their combinations were not evaluated because of very small sample size. Fifth, prognosis prediction analysis was not carried out in ICC or CHC because of small sample sizes. Sixth, HBV replication was not found to be significantly associated with the progression of ICC, which might also be related to insufficient power due to small sample size.

Conclusively, this large epidemiological study demonstrates that HBV infection contributes to the aggressiveness of HCC in China and possibly in other HBV-endemic areas. The contribution of HBV to the aggressiveness of ICC and other risk factors to the aggressiveness of HCC might be indirect *via* arousing non-resolving inflammation.

## Data Availability

All datasets generated for this study are included in the manuscript and/or the Supplementary Files.

## Ethics Statement

This study conformed to the ethical guidelines of the 1975 Declaration of Helsinki as reflected in a priori approval by the ethics committee of Eastern Hepatobiliary Surgery Hospital.

## Author Contributions

FY, LM, and WL contributed to the data organization, data analyses, and data interpretation. YY, JZ, MW, SC, and FS contributed to the patient enrolment, surgical treatment, and follow-up. XC, HZ, and HW conducted data collection and analyses. WZ performed surgical treatment as well as construction and maintenance of the database and BioBank. GC contributed to the study design, supervision, and writing of the manuscript.

### Conflict of Interest Statement

The authors declare that the research was conducted in the absence of any commercial or financial relationships that could be construed as a potential conflict of interest.

## Abbreviations

AFP: α-fetoprotein
BCLC: Barcelona Clinic Liver Cancer
CA19-9: carbohydrate antigen 19-9
CHC: combined hepatocellular cholangiocarcinoma
CI: confidence interval
HBV: hepatitis B virus
HBsAg: hepatitis B surface antigen
HCV: hepatitis C virus
HR: hazard ratio
HCC: hepatocellular carcinoma
ICC: intrahepatic cholangiocarcinoma
IQR: interquartile range
MVI: microvascular invasion
NLR: neutrophil-to-lymphocyte ratio
OS: overall survival
PLR: platelet-to-lymphocyte ratio
PLC: primary liver cancer
RFA: radiofrequency ablation
TACE: transcatheter arterial chemoembolization.
