# Supplementary material for: Contribution of Hepatitis B Virus Infection to the Aggressiveness of Primary Liver Cancer: A Clinical Epidemiological Study in Eastern China
Source: Front Oncol. 2019 May 21;9:370. doi: 10.3389/fonc.2019.00370 (PMC6537574; doi:10.3389/fonc.2019.00370)
Supplement: Supplementary file 1 [file Data_Sheet_1.docx]

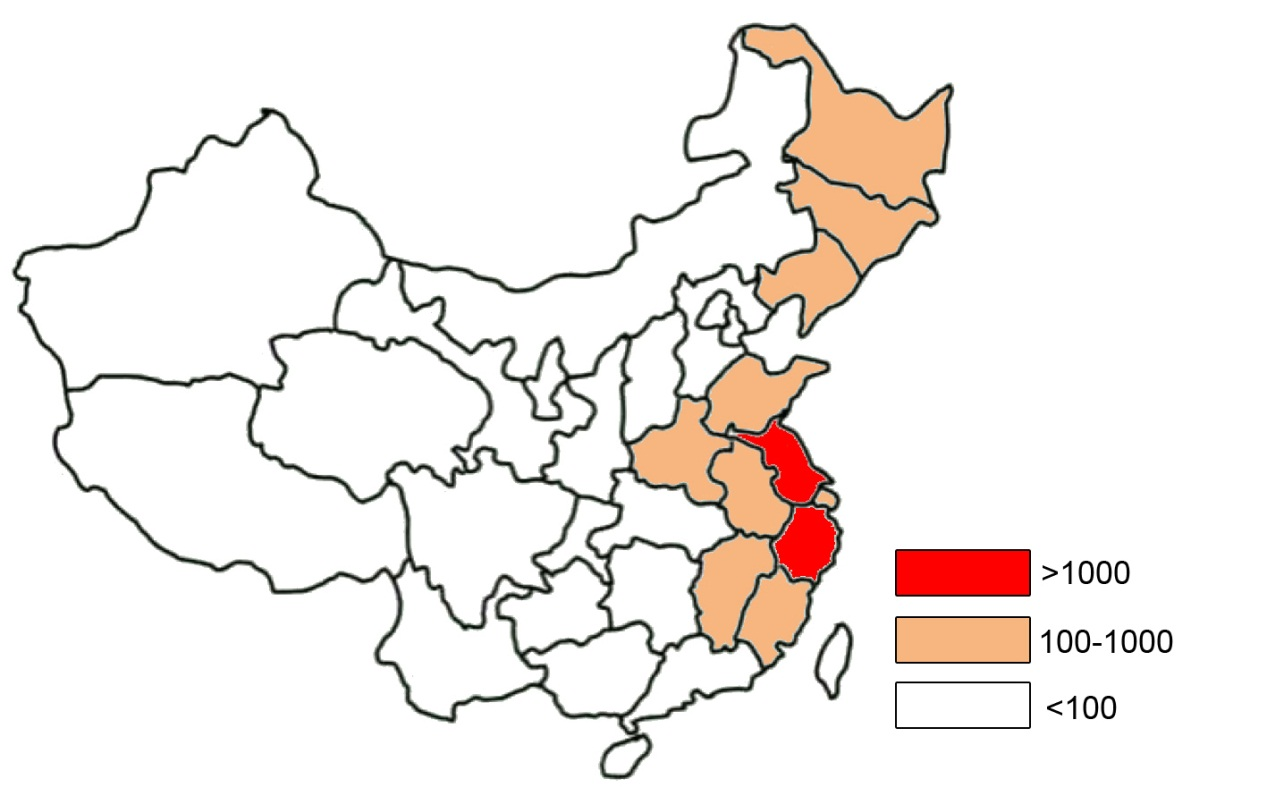


**Supplementary Figure 1. Geographic distribution of the 8515 included patients**


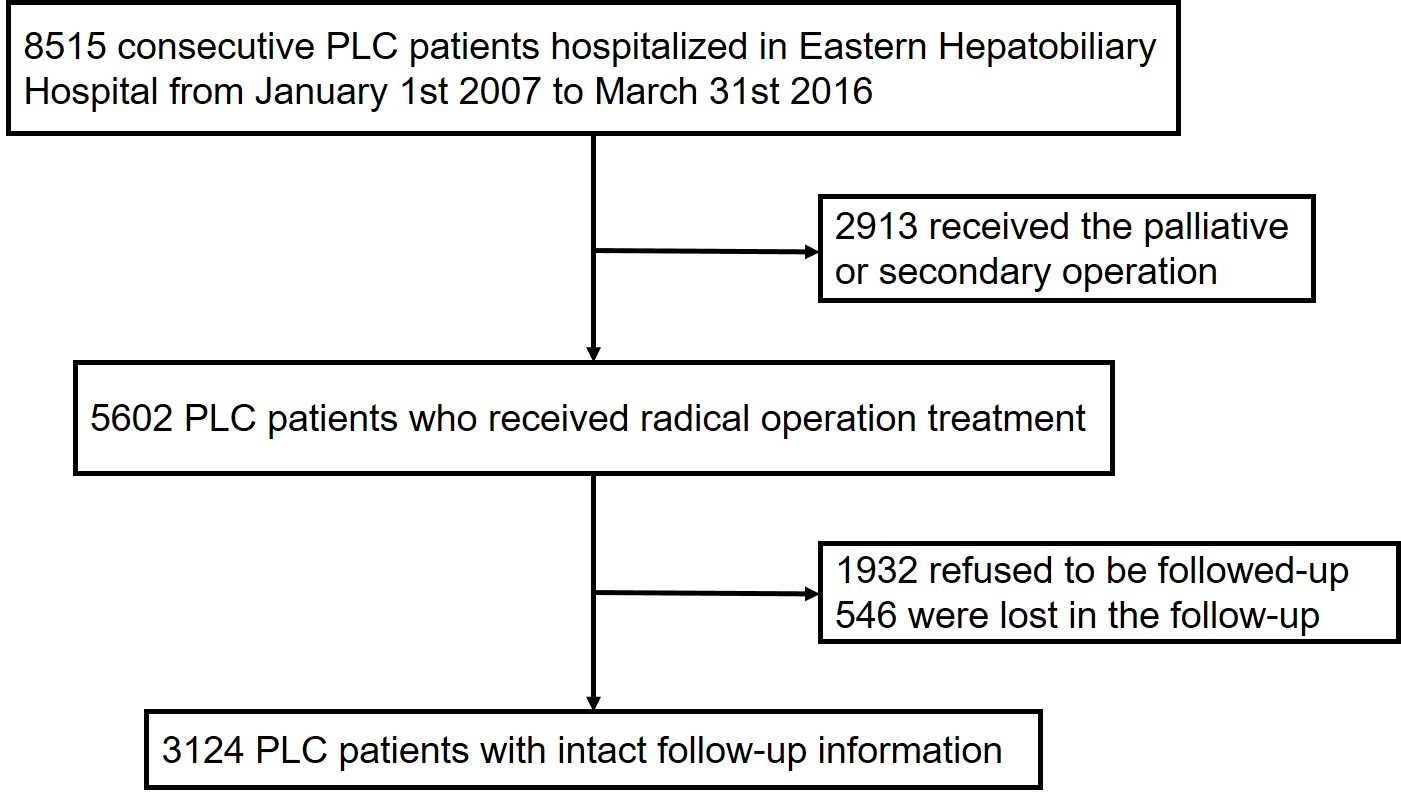


**Supplementary Figure 2. Flow diagram of participants.** PLC, primary liver cancer


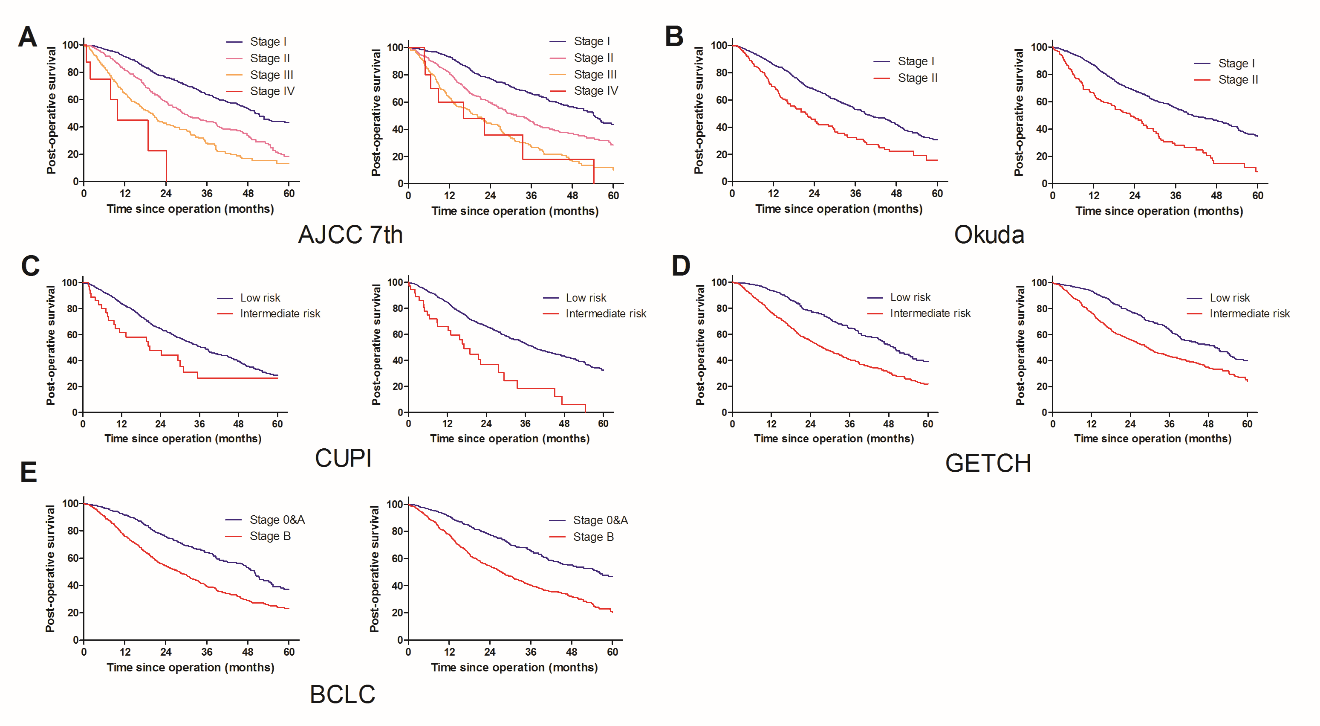


**Supplementary Figure 3. Kaplan-Meier survival curves of the training cohort and validation cohort**. (A) AJCC 7th; (B) Okuda; (C) CUPI; (D) GETCH; (E) BCLC. The left of each panel was for training cohort, while the right was for the validation cohort. AJCC, American Joint Committee on Cancer; CUPI, Chinese University Prognostic Index; GETCH, Groupe d’Etude et de Traitement du Carcinome Hepatocellulaire Prognostic scoreification; BCLC, Barcelona Clinic Liver Cancer; HCC, hepatocellular carcinoma.

**Supplementary Table 1. Baseline information of 8515 PLC patients and comparison of three histological types** ^a^

| Variable | All patients (N=8515) ^b^ | HCC (N=8056) ^b^ | ICC (N=314) ^b^ | CHC (N=145) ^b^ | Multi-group comparison | HCC *vs.* ICC | HCC *vs.* CHC | ICC *vs.* CHC |
| --- | --- | --- | --- | --- | --- | --- | --- | --- |
|  |  |  |  |  | *P* ^c^ | *P* ^c^ | *P* ^c^ | *P* ^c^ |
| Age, medium (IQR) | 54 (46-61) | 53 (46-61) | 58 (49-64) | 56 (46.5-64) | <0.001 | <0.001 | 0.035 | 0.104 |
| Gender |  |  |  |  |  |  |  |  |
| Ratio (M:F) | 6.1 | 6.6 | 2.2 | 4.6 |  |  |  |  |
| Male | 7324 (86.0) | 6990 (86.8) | 215 (68.5) | 119 (82.1) | <0.001 | <0.001 | 0.099 | 0.002 |
| Female | 1191 (14.0) | 1066 (13.2) | 99 (31.5) | 26 (17.9) |  |  |  |  |
| Cirrhosis (ultrasound) | |  |  |  |  |  |  |  |
| No | 4645 (57.8) | 4346 (57.1) | 217 (73.3) | 82 (59.4) | <0.001 | <0.001 | 0.588 | 0.004 |
| Yes | 3398 (32.2) | 3263 (42.9) | 79 (26.7) | 56 (40.6) |  |  |  |  |
| Cirrhosis (pathology) | |  |  |  |  |  |  |  |
| No | 4682 (55.2) | 4370 (54.4) | 229 (73.6) | 83 (57.6) | <0.001 | <0.001 | 0.444 | 0.001 |
| Yes | 3801 (44.8) | 3658 (45.6) | 82 (26.4) | 61 (42.4) |  |  |  |  |
| AFP (ng/mL) | |  |  |  |  |  |  |  |
| Medium | 86.5 | 101.6 | 4.1 | 40.6 |  |  |  |  |
| Negative (<20) | 3189 (38.0) | 2894 (36.4) | 239 (77.6) | 56 (39.4) | <0.001 | <0.001 | 0.458 | <0.001 |
| Positive (≥20) | 5209 (62.0) | 5054 (63.6) | 69 (22.4) | 86 (60.6) |  |  |  |  |
| CA19-9 (U/mL) | |  |  |  |  |  |  |  |
| Medium | 18 | 17.7 | 38.5 | 23.8 |  |  |  |  |
| Negative (<37) | 6376 (79.1) | 6140 (80.6) | 148 (49.7) | 88 (61.5) | <0.001 | <0.001 | <0.001 | 0.019 |
| Positive (≥37) | 1682 (20.9) | 1477 (19.4) | 150 (50.3) | 55 (38.5) |  |  |  |  |
| HBsAg |  |  |  |  |  |  |  |  |
| Negative | 1197 (14.2) | 1012 (12.7) | 157 (50.8) | 28 (19.4) | <0.001 | <0.001 | 0.017 | <0.001 |
| Positive | 7205 (85.8) | 6937 (87.3) | 152 (49.2) | 116 (80.6) |  |  |  |  |
| HBeAg |  |  |  |  |  |  |  |  |
| Negative | 6264 (74.6) | 5887 (74.1) | 264 (85.4) | 113 (78.5) | <0.001 | <0.001 | 0.231 | 0.065 |
| Positive | 2138 (25.4) | 2062 (25.9) | 45 (14.6) | 31 (21.5) |  |  |  |  |
| HBcAb |  |  |  |  |  |  |  |  |
| Negative | 190 (2.3) | 145 (1.8) | 41 (13.3) | 4 (2.8) | <0.001 | <0.001 | 0.596 | <0.001 |
| Positive | 8212 (97.7) | 7804 (98.2) | 268 (86.7) | 140 (97.2) |  |  |  |  |
| HBV DNA (copies/mL) | |  |  |  |  |  |  |  |
| Undetectable | 3958 (49.1) | 3703 (48.3) | 176 (66.2) | 79 (58.5) | <0.001 | <0.001 | 0.019 | 0.133 |
| Detectable | 4103 (50.9) | 3957 (51.7) | 90 (33.8) | 56 (41.5) |  |  |  |  |
| 500-1000000 | 2125 (26.4) | 2060 (26.9) | 45 (16.9) | 20 (14.8) |  |  |  |  |
| >1000000 | 1978 (24.5) | 1897 (24.8) | 45 (16.9) | 36 (26.7) |  |  |  |  |
| Anti-HCV |  |  |  |  |  |  |  |  |
| Negative | 7853 (98.3) | 7446 (98.3) | 268 (98.2) | 139 (98.6) | 0.953 |  |  |  |
| Positive | 137 (1.7) | 130 (1.7) | 5 (1.8) | 2 (1.4) |  |  |  |  |
| Hepatitis |  |  |  |  |  |  |  |  |
| HBV alone | 6757 (85.0) | 6519 (86.4) | 126 (46.5) | 112 (80.0) | <0.001 | <0.001 | 0.474 | <0.001 |
| HCV alone | 86 (1.1) | 81 (1.1) | 4 (1.5) | 1 (0.7) |  |  |  |  |
| Coinfection | 50 (0.6) | 48 (0.6) | 1 (0.4) | 1 (0.7) |  |  |  |  |
| No infection | 1059 (13.3) | 893 (11.8) | 140 (51.7) | 26 (18.6) |  |  |  |  |
| Total bilirubin (μmol/L) | |  |  |  |  |  |  |  |
| Medium (IQR) | 13.7 (10.6-17.8) | 13.8 (10.6-17.9) | 12.3 (9.2-16.2) | 13.1 (10.3-16.9) |  |  |  |  |
| ≤20 | 6977 (83.8) | 6588 (83.7) | 270 (87.4) | 119 (85.1) | 0.221 |  |  |  |
| >20 | 1345 (16.2) | 1284 (16.3) | 39 (12.6) | 22 (14.9) |  |  |  |  |
| Direct bilirubin (μmol/L) | |  |  |  |  |  |  |  |
| Medium (IQR) | 5.2 (4.0-6.9) | 5.3 (4.0-6.9) | 4.6 (3.5-6.3) | 5.0 (3.9-6.8) |  |  |  |  |
| ≤7 | 6360 (76.4) | 6002 (76.2) | 250 (80.9) | 108 (76.6) | 0.166 |  |  |  |
| >7 | 1962 (23.6) | 1870 (23.8) | 59 (19.1) | 33 (23.4) |  |  |  |  |
| Albumin (g/L) | |  |  |  |  |  |  |  |
| Medium (IQR) | 41.8 (39.3-44.3) | 41.8 (39.3-44.3) | 42.5 (40.2-44.9) | 42.0 (38.9-45.0) |  |  |  |  |
| >35 | 7779 (95.2) | 7357 (95.2) | 288 (96.3) | 134 (95.0) | 0.647 |  |  |  |
| ≤35 | 393 (4.8) | 375 (4.8) | 11 (3.7) | 7 (5.0) |  |  |  |  |
| NLR |  |  |  |  |  |  |  |  |
| Medium (IQR) | 2.0 (1.5-2.9) | 2.0 (1.5-2.8) | 2.5 (1.9-3.8) | 2.6 (1.7-3.5) |  |  |  |  |
| ≤3.3 | 6910 (81.3) | 6604 (82.2) | 204 (65.0) | 102 (70.3) | <0.001 | <0.001 | <0.001 | 0.256 |
| >3.3 | 1585 (18.7) | 1432 (17.8) | 110 (35.0) | 43 (29.7) |  |  |  |  |
| PLR |  |  |  |  |  |  |  |  |
| Medium (IQR) | 101.3 (75.7-138.0) | 100.6 (75.2-137.1) | 116.1 (88.9-162.9) | 111.8 (85.0-147.3) |  |  |  |  |
| ≤117 | 5347 (62.9) | 5111 (63.6) | 157 (50.0) | 79 (54.5) | <0.001 | <0.001 | 0.024 | 0.372 |
| >117 | 3148 (37.1) | 2925 (36.4) | 157 (50.0) | 66 (45.5) |  |  |  |  |
| Child-Pugh score |  |  |  |  |  |  |  |  |
| A | 7732 (98.8) | 7304 (98.8) | 292 (97.7) | 136 (99.3) | 0.172 |  |  |  |
| B | 95 (1.2) | 87 (1.2) | 7 (2.3) | 1 (0.7) |  |  |  |  |
| BCLC stage |  |  |  |  |  |  |  |  |
| 0 | 378 (4.5) | 370 (4.6) | 3 (1.0) | 5 (3.5) | <0.001 | <0.001 | 0.004 | 0.156 |
| A | 3028 (35.9) | 2922 (36.6) | 67 (22.0) | 39 (27.1) |  |  |  |  |
| B | 3699 (43.8) | 3469 (43.4) | 162 (53.1) | 68 (47.2) |  |  |  |  |
| C | 1336 (15.8) | 1231 (15.4) | 73 (23.9) | 32 (22.2) |  |  |  |  |
| Tumor diameter (cm) | |  |  |  |  |  |  |  |
| <3 | 1731 (20.6) | 1687 (21.1) | 25 (8.3) | 19 (13.3) | <0.001 | <0.001 | 0.022 | 0.101 |
| ≥3 | 6690 (79.4) | 6290 (78.9) | 276 (91.7) | 124 (86.7) |  |  |  |  |
| Tumor number | |  |  |  |  |  |  |  |
| Single | 6830 (81.2) | 6472 (81.2) | 251 (83.4) | 107 (74.8) | 0.093 |  |  |  |
| Multiple | 1583 (18.8) | 1497 (18.8) | 50 (16.6) | 36 (25.2) |  |  |  |  |
| Tumor encapsulation | |  |  |  |  |  |  |  |
| No | 2253 (26.8) | 1931 (24.2) | 256 (85.0) | 66 (47.5) | <0.001 | <0.001 | <0.001 | <0.001 |
| Yes | 6167 (73.2) | 6049 (75.8) | 45 (15.0) | 73 (52.5) |  |  |  |  |
| MVI |  |  |  |  |  |  |  |  |
| No | 5331 (63.1) | 5010 (62.6) | 244 (79.7) | 78 (55.7) | <0.001 | <0.001 | 0.095 | <0.001 |
| Yes | 3116 (36.9) | 2993 (37.4) | 62 (20.3) | 62 (44.3) |  |  |  |  |

^a^ Data are presented as number (%), unless otherwise indicated.

^b^ Some data do not sum up to the total number for existence of missing data. Some percentages do not sum up to 100 because of rounding.

^c^ For age (continuous variable) and BCLC stage (rank variable), Kruskal–Wallis ANOVA was conducted. For other variables (categorical variables), chi-square test was conducted. The Bonferroni correction was applied for multiple comparisons, and P value <0.0167 was considered significant.

PLC, primary liver cancer; HCC, Hepatocellular carcinoma; ICC, intrahepatic cholangiocarcinoma; CHC, combined hepatocellular cholangiocarcinoma; IQR, interquartile range; AFP, a-fetoprotein; CA19-9, carbohydrate antigen 19-9; HBsAg, hepatitis B surface antigen; HBeAg, hepatitis B e antigen; HBcAb, hepatitis B core antibody; HBV, hepatitis B virus; HCV, hepatitis C virus; NLR, neutrophil-lymphocyte ratio; PLR, platelet-lymphocyte ratio; BCLC, Barcelona Clinic Liver Cancer; MVI, microvascular invasion.

**Supplementary Table 2. Comparison of demographical and clinical characteristics between CHC patients with HBV infection and those without HBV infection**

| Variable | Patients without HBV infection (N=28) ^a^ | Patients with HBV infection (N=116) ^a^ | *P* ^b^ |
| --- | --- | --- | --- |
| Gender |  |  |  |
| Female | 6 (21.4) | 19 (16.4) | 0.722 |
| Male | 22 (78.6) | 97 (83.6) |  |
| Age |  |  |  |
| Medium (IQR) | 64 (60-65) | 53 (45-63) | <0.001 |
| ≤40 | 0 (0.0) | 13 (11.2) |  |
| 40-60 | 9 (32.1) | 69 (59.5) |  |
| >60 | 19 (67.9) | 34 (29.3) |  |
| Cirrhosis (ultrasound) |  |  |  |
| No | 22 (81.5) | 59 (53.6) | 0.008 |
| Yes | 5 (18.5) | 51 (46.4) |  |
| Cirrhosis (pathology) |  |  |  |
| No | 25 (89.3) | 57 (49.6) | <0.001 |
| Yes | 3 (10.7) | 58 (50.4) |  |
| AFP (ng/mL) |  |  |  |
| Negative (<20) | 17 (63.0) | 38 (33.3) | 0.004 |
| Positive (≥20) | 10 (37.0) | 76 (66.7) |  |
| CA19-9 (U/mL) |  |  |  |
| Negative (<37) | 18 (66.7) | 70 (60.3) | 0.543 |
| Positive (≥37) | 9 (33.3) | 46 (39.7) |  |
| HBeAg |  |  |  |
| Negative | 28 (100.0) | 85 (73.3) | 0.002 |
| Positive | 0 (0.0) | 31 (26.7) |  |
| HBcAb |  |  |  |
| Negative | 4 (14.3) | 0 (0.0) | <0.001 |
| Positive | 24 (85.7) | 116 (100.0) |  |
| Total bilirubin (μmol/L) |  |  |  |
| ≤20 | 23 (82.1) | 96 (85.7) | 0.859 |
| >20 | 5 (17.9) | 16 (14.3) |  |
| Direct bilirubin (μmol/L) |  |  |  |
| ≤7 | 23 (82.1) | 85 (75.9) | 0.481 |
| >7 | 5 (17.9) | 27 (24.1) |  |
| Albumin (g/L) |  |  |  |
| Medium (IQR) | 42.2 (39.2-43.7) | 41.9 (38.8-45.1) |  |
| >35 | 27 (96.4) | 106 (94.6) | 1.000 |
| ≤35 | 1 (3.6) | 6 (5.4) |  |
| NLR |  |  |  |
| Medium (IQR) | 3.2 (2.0-5.0) | 2.4 (1.6-3.3) |  |
| ≤3.3 | 14 (50.0) | 87 (75.0) | 0.009 |
| >3.3 | 14 (50.0) | 29 (25.0) |  |
| PLR |  |  |  |
| Medium (IQR) | 141.5 (110.0-188.6) | 104.4 (80.0-138.0) |  |
| ≤117 | 9 (32.1) | 69 (59.5) | 0.009 |
| >117 | 19 (67.9) | 47 (40.5) |  |
| BCLC stage |  |  |  |
| 0 | 0 (0.0) | 5 (4.3) | 0.458 |
| A | 8 (29.6) | 31 (26.7) |  |
| B | 11 (40.7) | 56 (48.3) |  |
| C | 8 (29.6) | 24 (20.7) |  |
| Child-Pugh score |  |  |  |
| A | 26 (100.0) | 109 (99.1) | 0.626 |
| B | 0 (0.0) | 1 (0.9) |  |
| Tumor diameter (cm) |  |  |  |
| <3 | 4 (14.8) | 15 (13.0) | 0.808 |
| ≥3 | 23 (85.2) | 100 (87.0) |  |
| Tumor number |  |  |  |
| Single | 19 (70.4) | 87 (75.7) | 0.570 |
| Multiple | 8 (29.6) | 28 (24.3) |  |
| Tumor encapsulation |  |  |  |
| No | 17 (63.0) | 49 (44.1) | 0.079 |
| Yes | 10 (37.0) | 62 (55.9) |  |
| MVI |  |  |  |
| No | 15 (57.7) | 62 (54.9) | 0.794 |
| Yes | 11 (42.3) | 51 (45.1) |  |

^a^ Data are presented as number (%), unless otherwise indicated. Some data do not sum up to the total number for the existence of missing data. Some percentages do not sum up to 100 because of rounding.

^b^ For age (continuous variable) and BCLC stage (rank variable), Mann-Whitney U test was conducted. For other variables (categorical variables), chi-square test was conducted.

CHC, combined hepatocellular cholangiocarcinoma; HBV, hepatitis B virus; IQR, interquartile range; AFP, a-fetoprotein; CA19-9, carbohydrate antigen 19-9; NLR, neutrophil-lymphocyte ratio; PLR, platelet-lymphocyte ratio; BCLC, Barcelona Clinic Liver Cancer; MVI, microvascular invasion

**Supplementary Table 3. Baseline characteristics of patients involved in survival analysis and those not.**

| Variable | Patients not involved in survival analysis | Patients involved in survival analysis | *P* |
| --- | --- | --- | --- |
|  | (N=2478) ^a^ | (N=3124) ^a^ |  |
| Age, medium (IQR) | 54 (47-62) | 54 (46-62) | 0.753 |
| Gender |  |  |  |
| Male | 2123 (85.7) | 2691 (86.1) | 0.619 |
| Female | 355 (14.3) | 433 (13.9) |  |
| Pathological type |  |  |  |
| HCC | 2357 (95.1) | 2963 (94.8) | 0.237 |
| ICC | 92 (3.7) | 108 (3.5) |  |
| CHC | 29 (1.2) | 53 (1.7) |  |
| Cirrhosis (ultrasound) | |  |  |
| No | 1384 (59.8) | 1708 (58.3) | 0.263 |
| Yes | 931 (40.2) | 1224 (41.7) |  |
| Cirrhosis (pathology) |  |  |  |
| No | 1443 (58.6) | 1750 (56.0) | 0.051 |
| Yes | 1018 (41.4) | 1373 (44.0) |  |
| AFP (ng/mL) |  |  |  |
| Negative (<20) | 1062 (43.3) | 1195 (38.8) | 0.001 |
| Positive (≥20) | 1391 (56.7) | 1882 (61.2) |  |
| CA19-9 (U/mL) |  |  |  |
| Negative (<37) | 1912 (81.2) | 2364 (79.9) | 0.227 |
| Positive (≥37) | 443 (18.8) | 596 (20.1) |  |
| HBsAg |  |  |  |
| Negative | 352 (14.4) | 443 (14.4) | 0.997 |
| Positive | 2097 (85.6) | 2640 (85.6) |  |
| HBeAg |  |  |  |
| Negative | 1831 (74.8) | 2296 (74.5) | 0.804 |
| Positive | 618 (25.2) | 787 (25.5) |  |
| HBcAb |  |  |  |
| Negative | 62 (2.5) | 64 (2.1) | 0.259 |
| Positive | 2387 (97.5) | 3019 (97.9) |  |
| HBV DNA |  |  |  |
| Undetectable | 1155 (49.0) | 1525 (51.4) | 0.079 |
| Detectable | 1204 (51.0) | 1443 (48.6) |  |
| anti-HCV |  |  |  |
| Negative | 2272 (98.5) | 2916 (98.1) | 0.220 |
| Positive | 34 (1.5) | 57 (1.9) |  |
| Total bilirubin (μmol/L) | |  |  |
| ≤20 | 2058 (84.5) | 2609 (85.7) | 0.226 |
| >20 | 378 (15.5) | 437 (14.3) |  |
| Direct bilirubin (μmol/L) | |  |  |
| ≤7 | 1886 (77.4) | 2368 (77.7) | 0.778 |
| >7 | 550 (22.6) | 678 (22.3) |  |
| Albumin (g/L) |  |  |  |
| >35 | 2311 (96.5) | 2862 (94.5) | <0.001 |
| ≤35 | 84 (3.5) | 168 (5.5) |  |
| NLR |  |  |  |
| ≤3.3 | 2063 (83.4) | 2587 (82.8) | 0.586 |
| >3.3 | 411 (16.6) | 536 (17.2) |  |
| PLR |  |  |  |
| ≤117 | 1636 (66.1) | 2010 (64.4) | 0.168 |
| >117 | 838 (33.9) | 1113 (35.6) |  |
| Child-Pugh score |  |  |  |
| A | 1948 (99.5) | 2823 (99.0) | 0.042 |
| B | 9 (0.5) | 28 (1.0) |  |
| BCLC stage |  |  |  |
| 0 | 140 (5.7) | 151 (4.9) | <0.001 |
| A | 1128 (46.2) | 1256 (40.4) |  |
| B | 1176 (48.1) | 1700 (54.7) |  |
| Tumor diameter (cm) |  |  |  |
| <3 | 613 (25.1) | 679 (21.9) | 0.005 |
| ≥3 | 1830 (74.9) | 2426 (78.1) |  |
| Tumor number |  |  |  |
| Single | 2089 (85.6) | 2456 (79.1) | <0.001 |
| Multiple | 352 (14.4) | 648 (20.9) |  |
| Tumor encapsulation |  |  |  |
| No | 636 (26.0) | 631 (20.3) | <0.001 |
| Yes | 1813 (74.0) | 2477 (79.7) |  |
| MVI |  |  |  |
| No | 1859 (75.7) | 2161 (69.6) | <0.001 |
| Yes | 597 (24.3) | 945 (30.4) |  |

^a^ Data are presented as number (%), unless otherwise indicated. Some percentages do not sum up to 100 because of rounding.

IQR, interquartile range; HCC, Hepatocellular carcinoma; ICC, intrahepatic cholangiocarcinoma; CHC, combined hepatocellular cholangiocarcinoma; AFP, alpha-fetoprotein; CA19-9, carbohydrate antigen 19-9; HBsAg, hepatitis B surface antigen; HBeAg, hepatitis B e antigen; HBcAb, hepatitis B core antibody; HBV, hepatitis B virus; HCV, hepatitis C virus; NLR, neutrophil-lymphocyte ratio; PLR, platelet-lymphocyte ratio; BCLC, Barcelona Clinic Liver Cancer; MVI, microvascular invasion.

**Supplementary Table 4. 1-, 3- and 5- year survival rates of HCC, ICC, and CHC patients**

|  |  | HCC | | |  | ICC | | |  | CHC | | |
| --- | --- | --- | --- | --- | --- | --- | --- | --- | --- | --- | --- | --- |
|  |  | ALL | Early stages | Advanced stages |  | ALL | Early stages | Advanced stages |  | ALL | Early stages | Advanced stages |
| 1-year |  | 83.4 | 91.2 | 76.8 |  | 47.3 | 58.3 | 42.9 |  | 72.4 | 89.1 | 64.2 |
| 3-year |  | 51.0 | 65.0 | 39.8 |  | 14.1 | 27.4 | 8.1 |  | 20.0 | 18.2 | 22.0 |
| 5-year |  | 30.1 | 42.0 | 21.7 |  | 7.8 | 27.4 | 1.6 |  | 13.3 | 18.2 | 11.0 |

HCC, Hepatocellular carcinoma; ICC, intrahepatic cholangiocarcinoma; CHC, combined hepatocellular cholangiocarcinoma.

**Supplementary Table 5. Univariate and multivariate Cox regression analysis of prognostic factors for post-operative survival in ICC patients**

| Variable | No. (%) of participants (n=2,963) ^a^ | Univariate analysis HR (95% CI) | *P* | Multivariate analysis HR (95% CI) ^b^ | *P* |
| --- | --- | --- | --- | --- | --- |
| Gender |  |  |  |  |  |
| Female | 38 (35.2) | 1 |  |  |  |
| Male | 70 (64.8) | 1.02 (0.66-1.57) | 0.945 |  |  |
| Age |  |  |  |  |  |
| <40 | 4 (3.7) | 1 |  |  |  |
| 40-59 | 63 (58.3) | 1.56 (0.48-5.00) | 0.458 |  |  |
| ≥60 | 41 (38.0) | 0.98 (0.30-3.20) | 0.969 |  |  |
| Cirrhosis (ultrasound) | |  |  |  |  |
| No | 75 (73.5) | 1 |  |  |  |
| Yes | 27 (26.5) | 0.97 (0.53-1.41) | 0.569 |  |  |
| Cirrhosis (pathology) | |  |  |  |  |
| No | 79 (73.1) | 1 |  |  |  |
| Yes | 29 (26.9) | 0.91 (0.56-1.46) | 0.69 |  |  |
| HBV DNA (copies/mL) | |  |  |  |  |
| <500 | 67 (74.4) | 1 |  |  |  |
| ≥500 | 23 (25.6) | 1.65 (0.95-2.88) | 0.076 |  |  |
| AFP (ng/mL) | |  |  |  |  |
| ≤20 | 82 (76.6) | 1 |  |  |  |
| >20 | 25 (23.4) | 1.35 (0.81-2.24) | 0.253 |  |  |
| CA19-9 (U/mL) | |  |  |  |  |
| ≤37 | 49 (47.1) | 1 |  | 1 |  |
| >37 | 55 (52.9) | 1.59 (1.03-2.44) | 0.035 | 1.67 (1.03-2.70) | 0.038 |
| HBsAg |  |  |  |  |  |
| Negative | 53 (50.0) | 1 |  |  |  |
| Positive | 53 (50.0) | 1.39 (0.91-2.13) | 0.123 |  |  |
| HBeAg |  |  |  |  |  |
| Negative | 89 (84.0) | 1 |  |  |  |
| Positive | 17 (16.0) | 1.22 (0.68-2.22) | 0.505 |  |  |
| Total bilirubin (μmol/L) | |  |  |  |  |
| ≤20 | 95 (88.0) | 1 |  |  |  |
| >20 | 13 (12.0) | 0.81 (0.43-1.54) | 0.523 |  |  |
| Direct bilirubin (μmol/L) | |  |  |  |  |
| ≤ 7 | 88 (81.5) | 1 |  |  |  |
| > 7 | 20 (18.5) | 1.15 (0.68-1.93) | 0.604 |  |  |
| Albumin (g/L) | |  |  |  |  |
| >35 | 104 (96.3) | 1 |  |  |  |
| ≤35 | 4 (3.7) | 0.44 (1.08-1.80) | 0.254 |  |  |
| NLR |  |  |  |  |  |
| ≤3.3 | 74 (68.5) | 1 |  |  |  |
| >3.3 | 34 (31.5) | 0.94 (0.60-1.47) | 0.769 |  |  |
| PLR |  |  |  |  |  |
| ≤117 | 61 (56.5) | 1 |  |  |  |
| >117 | 47 (43.5) | 1.19 (0.78-1.81) | 0.421 |  |  |
| Tumor diameter | |  |  |  |  |
| <3 | 7 (6.5) | 1 |  |  |  |
| ≥3 | 100 (93.5) | 0.70 (0.32-1.53) | 0.376 |  |  |
| Tumor number | |  |  |  |  |
| Single | 86 (80.4) | 1 |  |  |  |
| Multiple | 21 (19.6) | 1.88 (1.12-3.15) | 0.017 |  |  |
| Tumor encapsulation | |  |  |  |  |
| No | 100 (92.6) | 1 |  |  |  |
| Yes | 8 (7.4) | 0.44 (0.18-1.08) | 0.073 |  |  |
| Child-Pugh score | |  |  |  |  |
| A | 108 (100) | 1 |  |  |  |
| B | 0 (0.0) | - | - |  |  |
| BCLC stage |  |  |  |  |  |
| 0&A | 32 (29.9) | 1 |  | 1 |  |
| B | 75 (70.1) | 1.94 (1.18-3.17) | 0.009 | 2.33 (1.32-4.09) | 0.003 |
| MVI |  |  |  |  |  |
| No | 93 (86.9) | 1 |  |  |  |
| Yes | 14 (13.1) | 1.28 (0.64-2.56) | 0.488 |  |  |
| post-operative TACE | |  |  |  |  |
| No | 72 (66.7) | 1 |  |  |  |
| Yes | 36 (33.3) | 0.71 (0.46-1.11) | 0.712 |  |  |
| Reoperation |  |  |  |  |  |
| No | 105 (97.2) | 1 |  |  |  |
| Yes | 3 (2.8) | 0.27 (0.04-1.92) | 0.190 |  |  |
| post-operative RFA | |  |  |  |  |
| No | 104 (96.3) | 1 |  |  |  |
| Yes | 4 (3.7) | 0.54 (0.17-1.71) | 0.295 |  |  |

^a^ Some data do not sum up to the total number for the existence of missing data.

^b^ The final model selection was carried out by a backward stepwise selection procedure with the Akaike information criterion. Only significant (*P*<0.05) covariates in univariate analysis were included.

ICC, intrahepatic cholangiocarcinoma; HR, hazard ratio; CI, confidence interval; HBV, hepatitis B virus; AFP, alpha-fetoprotein; CA19-9, carbohydrate antigen 19-9; HBsAg, hepatitis B surface antigen; HBeAg, hepatitis B e antigen; anti-HCV, hepatitis C virus antibody; NLR, neutrophil-lymphocyte ratio; PLR, platelet-lymphocyte ratio; BCLC, Barcelona Clinic Liver Cancer; MVI, microvascular invasion; TACE, transarterial chemoembolization; RFA, radiofrequency ablation.

**Supplementary Table 6. Univariate and multivariate Cox regression analysis of prognostic factors for postoperative survival in HCC patients seropositive for HBsAg**

| Variable | Univariate analysis HR (95% CI) | *P* | Multivariate analysis HR (95% CI) ^a^ | *P* |
| --- | --- | --- | --- | --- |
|  |  |  |  |  |
| Gender |  |  |  |  |
| Female | 1 |  |  |  |
| Male | 1.16 (0.95-1.41) | 0.141 |  |  |
| Age |  |  |  |  |
| <40 | 1 |  |  |  |
| 40-59 | 0.89 (0.73-1.08) | 0.231 |  |  |
| ≥60 | 0.88 (0.71-1.09) | 0.248 |  |  |
| Cirrhosis (ultrasound) |  |  |  |  |
| No | 1 |  |  |  |
| Yes | 1.02 (0.89-1.16) | 0.800 |  |  |
| Cirrhosis (pathology) |  |  |  |  |
| No | 1 |  |  |  |
| Yes | 1.02 (0.90-1.16) | 0.758 |  |  |
| AFP (ng/mL) |  |  |  |  |
| ≤20 | 1 |  | 1 |  |
| >20 | 1.98 (1.71-2.29) | <0.001 | 1.64 (1.40-1.93) | <0.001 |
| CA19-9 (U/mL) |  |  |  |  |
| ≤37 | 1 |  | 1 |  |
| >37 | 1.30 (1.12-1.51) | 0.001 | 1.27 (1.07-1.49) | 0.005 |
| HBV DNA |  |  |  |  |
| Undetectable | 1 |  | 1 |  |
| Detectable | 1.53 (1.34-1.75) | <0.001 | 1.35 (1.17-1.56) | <0.001 |
| HBeAg |  |  |  |  |
| Negative | 1 |  |  |  |
| Positive | 1.16 (1.02-1.33) | 0.028 |  |  |
| anti-HCV |  |  |  |  |
| Negative | 1 |  |  |  |
| Positive | 1.46 (0.69-3.07) | 0.319 |  |  |
| Total bilirubin (μmol/L) |  |  |  |  |
| ≤20 | 1 |  |  |  |
| >20 | 1.20 (1.01-1.42) | 0.040 |  |  |
| Direct bilirubin (μmol/L) |  |  |  |  |
| ≤ 7 | 1 |  |  |  |
| > 7 | 1.16 (1.00-1.34) | 0.050 |  |  |
| Albumin (g/L) |  |  |  |  |
| >35 | 1 |  |  |  |
| ≤35 | 1.23 (0.95-1.58) | 0.113 |  |  |
| NLR |  |  |  |  |
| ≤3.3 | 1 |  | 1 |  |
| >3.3 | 1.55 (1.32-1.82) | <0.001 | 1.39 (1.16-1.67) | <0.001 |
| PLR |  |  |  |  |
| ≤117 | 1 |  |  |  |
| >117 | 1.44 (1.27-1.63) | <0.001 |  |  |
| Tumor diameter (cm) |  |  |  |  |
| <3 | 1 |  | 1 |  |
| ≥3 | 2.15 (1.80-2.58) | <0.001 | 1.47 (1.16-1.87) | 0.002 |
| Tumor number |  |  |  |  |
| Single | 1 |  | 1 |  |
| Multiple | 1.63 (1.42-1.87) | <0.001 | 1.26 (1.08-1.48) | 0.004 |
| Tumor encapsulation |  |  |  |  |
| No | 1 |  | 1 |  |
| Yes | 0.64 (0.55-0.74) | <0.001 | 0.65 (0.55-0.76) | <0.001 |
| Child-Pugh score |  |  |  |  |
| A | 1 |  |  |  |
| B | 2.00 (1.22-3.29) | 0.006 |  |  |
| BCLC stage |  |  |  |  |
| 0&A | 1 |  | 1 |  |
| B | 2.16 (1.89-2.47) | <0.001 | 1.49 (1.24-1.80) | <0.001 |
| MVI |  |  |  |  |
| No | 1 |  | 1 |  |
| Yes | 2.13 (1.88-2.42) | <0.001 | 1.72 (1.49-1.97) | <0.001 |
| Post-operative TACE |  |  |  |  |
| No | 1 |  |  |  |
| Yes | 1.18 (1.04-1.34) | 0.010 |  |  |
| Reoperation |  |  |  |  |
| No | 1 |  | 1 |  |
| Yes | 0.51 (0.39-0.67) | <0.001 | 0.52 (0.38-0.71) | <0.001 |
| Post-operative RFA |  |  |  |  |
| No | 1 |  | 1 |  |
| Yes | 0.66 (0.53-0.82) | <0.001 | 0.66 (0.52-0.83) | <0.001 |

^a^ The final model selection was carried out by a backward stepwise selection procedure with the Akaike information criterion. Only significant (*P*<0.05) covariates in univariate analysis were included.

HBsAg, hepatitis B surface antigen; HR, hazard ratio; CI, confidence interval; HBV, hepatitis B virus; AFP, alpha fetoprotein; CA19-9, carbohydrate antigen 19-9; HBeAg, hepatitis B e antigen; HBcAb, hepatitis B core antibody; anti-HCV, hepatitis C virus antibody; NLR, neutrophil to lymphocyte ratio; PLR, platelet to lymphocyte ratio; BCLC, Barcelona Clinic Liver Cancer; MVI, microvascular invasion; TACE, transarterial chemoembolization; RFA, radiofrequency ablation.

**Supplementary Table 7. Comparison of demographical and clinical characteristics between training cohort and validation cohort**

| Variable | Training cohort (N=1482) | Validation cohort (N=1481) | *P* |
| --- | --- | --- | --- |
| Gender |  |  |  |
| Ratio (M:F) | 6.3 | 7.3 |  |
| Female | 203 (13.7) | 179 (12.1) | 0.207 |
| Male | 1279 (86.3) | 1302 (87.9) |  |
| Age |  |  |  |
| Medium (IQR) | 54 (46-62) | 53 (46-62) | 0.529 |
| ≤40 | 155 (10.5) | 169 (11.4) |  |
| 40-60 | 920 (62.1) | 880 (59.4) |  |
| >60 | 407 (27.5) | 432 (29.2) |  |
| Cirrhosis (ultrasound) | |  |  |
| No | 810 (58.4) | 792 (56.7) | 0.378 |
| Yes | 577 (41.6) | 604 (43.3) |  |
| Cirrhosis (pathology) | |  |  |
| No | 819 (55.3) | 824 (55.7) | 0.825 |
| Yes | 663 (44.7) | 656 (44.3) |  |
| AFP (ng/mL) | |  |  |
| Negative (<20) | 532 (36.5) | 560 (38.4) | 0.302 |
| Positive (≥20) | 926 (63.5) | 900 (61.6) |  |
| CA19-9 (U/mL) | |  |  |
| Negative (<37) | 1122 (79.9) | 1162 (83.0) | 0.033 |
| Positive (≥37) | 283 (20.1) | 238 (17.0) |  |
| HBsAg |  |  |  |
| Negative | 191 (13.1) | 191 (13.1) | 1.000 |
| Positive | 1272 (86.9) | 1271 (86.9) |  |
| HBeAg |  |  |  |
| Negative | 1080 (73.8) | 1090 (74.6) | 0.673 |
| Positive | 383 (26.2) | 372 (25.4) |  |
| HBcAb |  |  |  |
| Negative | 18 (1.2) | 30 (2.1) | 0.083 |
| Positive | 1445 (98.8) | 1432 (97.9) |  |
| HBV DNA (copies/mL) | |  |  |
| Undetectable (<500) | 693 (49.1) | 740 (52.1) | 0.114 |
| Detectable (≥500) | 718 (50.9) | 679 (47.9) |  |
| Total bilirubin (μmol/L) | |  |  |
| Medium (IQR) | 13.4 (10.4-17.5) | 13.5 (10.5-17.3) |  |
| ≤20 | 1227 (85.0) | 1240 (86.1) | 0.397 |
| >20 | 217 (15.0) | 200 (13.9) |  |
| Direct bilirubin (μmol/L) | |  |  |
| Medium (IQR) | 5.2 (4.0-6.9) | 5.2 (4.0-6.7) |  |
| ≤7 | 1105 (76.5) | 1132 (78.6) | 0.181 |
| >7 | 339 (23.5) | 308 (21.4) |  |
| Albumin (g/L) | |  |  |
| Medium (IQR) | 42.3 (40.0-45.0) | 41.8 (39.2-44.2) |  |
| >35 | 1352 (94.2) | 1355 (94.6) | 0.746 |
| ≤35 | 83 (5.8) | 78 (5.4) |  |
| NLR |  |  |  |
| Medium (IQR) | 1.9 (1.5-2.7) | 2.0 (1.5-2.7) |  |
| ≤3.3 | 1234 (83.3) | 1243 (84.0) | 0.620 |
| >3.3 | 248 (16.7) | 237 (16.0) |  |
| PLR |  |  |  |
| Medium (IQR) | 98.1 (75.1-133.3) | 99.7 (74.7-136.5) |  |
| ≤117 | 965 (65.1) | 950 (64.2) | 0.617 |
| >117 | 517 (34.9) | 530 (35.8) |  |
| Tumor diameter (cm) | |  |  |
| Medium (IQR) | 5.0 (2.5-8.0) | 5.0 (2.5-8.0) | 0.454 |
| <3 | 324 (22.0) | 341 (23.2) |  |
| ≥3 | 1151 (78.0) | 1131 (76.8) |  |
| Tumor number | |  |  |
| Single | 1170 (79.3) | 1169 (79.4) | 0.964 |
| Multiple | 305 (20.7) | 303 (20.6) |  |
| Tumor encapsulation | |  |  |
| No | 261 (17.6) | 262 (17.7) | 0.962 |
| Yes | 1221 (82.4) | 1219 (82.3) |  |
| BCLC stage |  |  |  |
| 0&A | 662 (44.9) | 693 (47.1) | 0.237 |
| B | 693 (55.1) | 2558 (52.9) |  |
| MVI |  |  |  |
| No | 1019 (69.2) | 1018 (69.0) | 0.936 |
| Yes | 454 (30.8) | 457 (31.0) |  |

IQR, interquartile range; AFP, alpha fetoprotein; HBsAg, hepatitis B surface antigen; HBeAg, hepatitis B e antigen; HR, hazard ratio; CI, confidence interval; HBV, hepatitis B virus; CA19-9, carbohydrate antigen 19-9; HBcAb, hepatitis B core antibody; anti-HCV, hepatitis C virus antibody; NLR, neutrophil to lymphocyte ratio; PLR, platelet to lymphocyte ratio; BCLC, Barcelona Clinic Liver Cancer; MVI, microvascular invasion.

**Supplementary Table 8. Univariate and multivariate Cox regression analysis of preoperative factors for postoperative survival in the training cohort (n=1482) ^a^**

| Variable | Univariate analysis | | Multivariate analysis ^b^ | |
| --- | --- | --- | --- | --- |
|  | HR (95% CI) | *P* | HR (95% CI) | *P* |
| Gender |  |  |  |  |
| Female | 1 |  |  |  |
| Male | 1.25 (0.96-1.62) | 0.098 |  |  |
| Age |  |  |  |  |
| <40 | 1 |  |  |  |
| 40-59 | 0.91 (0.69-1.20) | 0.517 |  |  |
| ≥60 | 0.90 (0.67-1.21) | 0.502 |  |  |
| Ultrasound cirrhosis |  |  |  |  |
| No | 1 |  | 1 |  |
| Yes | 1.23 (1.04-1.45) | 0.019 | 1.28 (1.07-1.55) | 0.008 |
| Serum AFP (ng/mL) |  |  |  |  |
| ≤20 | 1 |  | 1 |  |
| 20-400 | 1.47 (1.17-1.85) | 0.001 | 1.42 (1.10-1.84) | 0.008 |
| >400 | 2.50 (2.05-3.06) | <0.001 | 2.27 (1.81-2.85) | <0.001 |
| HBsAg |  |  |  |  |
| Negative | 1 |  |  |  |
| Positive | 1.46 (1.12-1.91) | 0.006 |  |  |
| HBeAg |  |  |  |  |
| Negative | 1 |  |  |  |
| Positive | 1.18 (0.99-1.42) | 0.067 |  |  |
| BCLC stage |  |  |  |  |
| 0&A | 1 |  | 1 |  |
| B | 2.05 (1.72-2.44) | <0.001 | 1.38 (1.07-1.78) | 0.012 |
| Log10 CA19-9 (U/mL) | 1.18 (0.98-1.41) | 0.083 |  |  |
| Log10 HBV-DNA | 1.14 (1.07-1.21) | <0.001 | 1.09 (1.02-1.17) | 0.015 |
| Total bilirubin (μmol/L) | 1.00 (0.98-1.01) | 0.737 |  |  |
| Direct bilirubin (μmol/L) | 1.00 (0.97-1.03) | 0.937 |  |  |
| Albumin (g/L) | 0.97 (0.95-0.99) | 0.004 |  |  |
| NLR | 1.01 (0.99-1.04) | 0.298 |  |  |
| PLR | 1.002 (1.001-1.004) | 0.002 |  |  |
| Tumor diameter (cm) | 1.09 (1.07-1.11) | <0.001 | 1.06 (1.03-1.09) | <0.001 |

^a^ Numbers of patients without intact information of cirrhosis, AFP, HBsAg, HBeAg, BCLC stage, CA19-9, HBV-DNA, total Bilirubin, direct Bilirubin, albumin, NLR, PLR, and tumor diameter were 95, 24, 19,19, 7, 85, 78, 61, 60, 69, 20, 18, and 11, respectively.

^b^ The final model selection was carried out by a backward stepwise selection procedure with the Akaike information criterion. Only significant (*P*<0.05) covariates in univariate analysis were included.

HR, hazard ratio; CI, confidence interval; AFP, alpha-fetoprotein; HBsAg, hepatitis B surface antigen; HBeAg, hepatitis B e antigen; BCLC, Barcelona Clinic Liver Cancer; CA19-9, carbohydrate antigen 19-9; HBV, hepatitis B virus; NLR, neutrophil to lymphocyte ratio; PLR, platelet to lymphocyte ratio.
